# Aniracetam Does Not Alter Cognitive and Affective Behavior in Adult C57BL/6J Mice

**DOI:** 10.1371/journal.pone.0104443

**Published:** 2014-08-06

**Authors:** Thomas W. Elston, Ashvini Pandian, Gregory D. Smith, Andrew J. Holley, Nanjing Gao, Joaquin N. Lugo

**Affiliations:** 1 Department of Psychology and Neuroscience, Baylor University, Waco, Texas, United States of America; 2 Institute of Biomedical Studies, Baylor University, Waco, Texas, United States of America; University of Akron, United States of America

## Abstract

There is a growing community of individuals who self-administer the nootropic aniracetam for its purported cognitive enhancing effects. Aniracetam is believed to be therapeutically useful for enhancing cognition, alleviating anxiety, and treating various neurodegenerative conditions. Physiologically, aniracetam enhances both glutamatergic neurotransmission and long-term potentiation. Previous studies of aniracetam have demonstrated the cognition-restoring effects of acute administration in different models of disease. No previous studies have explored the effects of aniracetam in healthy subjects. We investigated whether daily 50 mg/kg oral administration improves cognitive performance in naïve C57BL/6J mice in a variety of aspects of cognitive behavior. We measured spatial learning in the Morris water maze test; associative learning in the fear conditioning test; motor learning in the accelerating rotarod test; and odor discrimination. We also measured locomotion in the open field test, anxiety through the elevated plus maze test and by measuring time in the center of the open field test. We measured repetitive behavior through the marble burying test. We detected no significant differences between the naive, placebo, and experimental groups across all measures. Despite several studies demonstrating efficacy in impaired subjects, our findings suggest that aniracetam does not alter behavior in normal healthy mice. This study is timely in light of the growing community of healthy humans self-administering nootropic drugs.

## Introduction

There is increasing nonmedical use of prescription stimulants for cognitive enhancement. In 2000 the use of nonprescription use of methylphenidate hydrochloride in adolescents and young adults was 1.2% and the rate increased to 2% in 2006 [1]. The rate of lifetime use of these drugs is reported to be 1.5%. Another report found that approximately 25% of colleges surveyed had a prevalence rate of 10% or higher for the nonmedical use of prescription opioids the prior year [2]. The use of prescription drugs for neuroenhancement has led Duke University to introduce a policy that does not allow for the nonmedical use of prescription stimulants, such as Adderall for academic purposes [3]. Duke University will treat such use as cheating. One consequence is that with such policies in place students may turn to other drugs that claim to be cognitive enhancers. Indeed, a quick search of the search word “Nootropic” will result in over 25,000 articles in the PubMed database. Even though the purpose of many of these drugs is to treat neurological disorders, some are being investigated for their use as a cognitive enhancer in healthy individuals. One compound that has received significant investigation is Aniracetam 1-[(4-methoxyphenyl)]-2-pyrrolidinone. Aniracetam is currently marketed on several websites with the claim to enhance cognitive performance.

Aniracetam is a piracetam analog that has few reported side effects [4]. Several studies provide evidence that aniracetam can improve cognitive performance. Aniracetam improves visual recognition, motor performance, and general intellectual function in humans [5]. Another report found that aniracetam improves memory in humans that have cognitive impairment [6]. Similar results of the cognitive enhancing benefits of aniracetam have been found using non-human animals [7]–[11]. In addition to the improvement in cognitive function, aniracetam reduces anxiety in mice [12]. This study found that aniracetam reduces anxiety across three different anxiety tests. The studies above are often referred to when aniracetam is marketed through various websites.

One caveat in many of the studies is that the improvement in cognition is found in subjects that had experimentally-induced cognitive impairment. One study used a N-methyl-D-aspartate (NMDA) receptor antagonist to induce cognitive impairment, which was then reversed by intrahippocampal injection of 10 µg/side of aniracetam [10]. Aniracetam decreases working memory errors and total errors in the radial arm maze, which measures working memory in rodents. There was also an increase in hippocampal theta power in the aniracetam-treated rats. However, there was no additional improvement in either measure in rats given aniracetam alone. In a separate study, aniracetam improved fear conditioning contextual memory in DBA/2J mouse strain but not in C57BL/6 mouse strain with a single dose of 100 mg/kg of aniracetam [11]. The DBA/2J strain has been shown to be a poorer performer than the C57BL/6 mice in spatial learning [13]. Therefore, improvement in learning only appears to occur in a strain that is a poor performer while no effect is found in a mouse strain that is not learning impaired.

The studies above support the use of aniracetam to improve performance in conditions where there is cognitive impairment. However, there is less evidence that aniracetam improves cognitive behavior in normal animals. In the studies reported here, we administered daily doses of 50 mg/kg aniracetam to mice to determine whether the drug impacts locomotion, anxiety, spatial learning, motor learning, and associative learning in the C57BL/6J mouse strain. In addition, we administered aniracetam through voluntary oral consumption to more closely mimic how humans would consume the substance. We used the 50 mg/kg dose since it is within the therapeutic range to impact anxiety and learning and memory [7], [12]. If aniracetam is a cognitive enhancer of normal function then we hypothesize that drug-treated animals would show improvement in learning tasks. However, our studies did not provide evidence that aniracetam treatment improves cognitive performance or alters a number of behaviors in normal healthy mice.

## Materials and Methods

### Ethics statement

This study was carried out in strict accordance with the recommendations in the Guide for the Care and Use of Laboratory Animals of the National Institutes of Health. The protocol was approved by Baylor University Institutional Care and Use Committee (Animal Assurance Number A3948–01).

### Animals

Thirty adult male C57BL/6J mice between the ages of 3 and 6 months old at the start of experimentation were used in these experiments. Subjects were bred at Baylor University. Subjects were trained and tested in two groups of 15, staggering testing of the groups by one week. Subjects were handled for approximately 90 seconds per day for five days to acclimate them to handing. Animals were individually housed in typical mouse cages with wood shavings in a humidity and temperature-controlled vivarium. A 12 hour light/dark cycle was maintained. All testing was performed in accordance and with approval from the Baylor University Institutional Care and Use Committee.

### Aniracetam treatment

Aniracetam (1-(4-Methoxybenzoyl)-2-pyrrolidinone) was obtained from Shanghai Soyoung Biotechnologies Inc. (Shanghai, China). In order to simulate human consumption of aniracetam, aniracetam was administered orally at 50 mg/kg in a mixture of 0.72% sucrose and 3% gelatin (St. Louis, MO, USA) matrix; the placebo group received a 0.72% sucrose and 3% gelatin matrix with no aniracetam, and the naïve control group received only an empty weigh boat. Sucrose was used to ensure consumption of the gelatin matrix. The gelatin matrix was prepared fresh twice each week and animals were weighed at the beginning of the week to account for weight changes and to maintain precise dosing throughout testing. Animals were given their dose of vehicle or aniracetam daily for 5 days per week. Animals were dosed one hour before testing each day and at the same time each day. This regimen allowed the animals thirty minutes to consume the gelatin and then thirty minutes to rest once transported from the vivarium to the testing rooms. The consumption of the gelatin was verified before testing by the experimenter. The experimenter removed the weigh boat and examined the cage to determine whether the subject consumed the drug or vehicle each day of testing. All testing was conducted within a three-hour therapeutic window of administration, as determined by a previous pharmacokinetic investigation of aniracetam [14]. We utilized a double-blind experimental design for the aniracetam treatment.

### Behavior training and testing

All 30 mice were tested on four learning tasks, two tasks which measure anxiety, and one measure of repetitive behavior. After the completion of each task, animals received one day of rest. Each task required one-to-five days of testing; therefore, the entire testing schedule was completed in six weeks. With the exception of marble-burying, rotarod, and odor discrimination, the performance of all animals was video-recorded and analyzed with video tracking software. Different experimenters were responsible for training and testing each group of animals, with one beginning one week after the other. No experimenter was aware of animals’ performance on other tasks until the entire battery of behavioral tests, for both testing cohorts, was completed. Before and after each testing session, apparatuses were cleaned with a 30% isopropanol solution. All animals were tested during the middle seven hours of the light cycle to ensure the time of day did not affect performance. All animals were euthanized at the conclusion of the experiment by decapitation.

#### Open-Field Exploration

The open-field test was used to measure locomotion and to measure anxiety. Animals were placed in the center of a 40 cm×40 cm×40 cm clear acrylic container box in a well-lit room with consistent lighting and noise. Their behavior was video-monitored via Noldus Ethovision XT (Noldus, The Netherlands) motion-tracking software for 10 minutes. We examined the time the animal spent in the outer and inner zones as a measure of anxiety.

#### Elevated-Plus Maze

The elevated plus maze was used to examine anxiety. The maze consists of four elevated arms (40 cm from floor). There were 2 sets of equidistant arms that are 65 cm long and 5cm wide. Two of the opposing arms were open and two opposing arms were enclosed by 15 cm high walls. The apparatus was constructed of white acrylic and located in a low light level room with constant background noise. Mice were placed in one of the open arms near the center of the maze at the beginning of the trial. The animals’ movement was monitored via motion-tracking software for 10 minutes. We used a separate video capturing system to record the videos to be scored at a later time. Time spent in the enclosed arms is associated with anxious behavior and time spent in the open arms is associated with non-anxious behavior.

#### Morris Water Maze

The Morris Water Maze was used to measure spatial navigation. This task was first described in 1982 by R. Morris et al. where the investigators lesioned the hippocampus of female rats and found significant impairment in the spatial navigation task across 30 trials [15]. The rats without a hippocampus had no difficulty in finding a visible platform (cue navigation) across 13 trials. Therefore, the phase of the test which involves the rodent using spatial navigation to find the hidden platform is believed to involve the hippocampus. For our study, we used modified methods for mice that were previously described [16] where animals were placed in a round pool (diameter ≈130cm, height ≈60 cm) of opaque water from which they can escape onto a submerged, hidden platform. The pool was located in a well-lit room with constant light and noise and had extra-maze spatial landmarks (i.e. multicolored geometric shapes) placed in consistent locations on the walls. The round pool was filled to within 18 cm of the top with water made opaque by the addition of non-toxic, water soluble paint. The maze was divided into four quadrants defined by the cardinal directions (i.e. North, East, South, West); a hidden platform was submerged 2 cm beneath the water’s surface and located in the same quadrant of the water maze. The animal had a maximum time of 1 minute to find the submerged platform. If the animal did not escape the maze via the hidden platform, then the animal was placed on the submerged platform for 10 seconds. If the animal successfully escaped the maze, it was allowed to remain on the platform for 10 seconds before being removed for the next trial. Each animal received 4 trials per block, two blocks per day, with four days of testing. On day five we conducted a visible platform trial. We placed a visible platform in two regions of the maze which had not previously contained the hidden platform. We then measured the latency to the visible platform for all animals. We conducted 2 trials per block across 2 blocks for a total of 4 trials. This visible platform test was conducted to determine motor deficits or visual deficits in the mice. All swim behavior was tracked via Noldus Ethovision XT motion-tracking software.

#### Rotarod

We used an accelerating rotarod test (Series 8 Rotorod; IITC Inc., Woodland Hills, CA, USA) in order to examine cerebellar motor memory. In this experiment, animals were placed on a rotating rod which gradually rotated from 5 to 40 rpm over a five minute trial. Animals underwent two trials per day with a 60 min intertrial interval (ITI) between each trial across four days of testing. The experimenter live-scored the quantity of time each animal was able to stay on the rotating rod before falling off.

#### Odor Discrimination

Adapting a three-day protocol developed previously [17], mice learned to navigate a square field in which unique odor-marked (e.g. coffee, almond, banana) food cups were located in three corners. We used a clear acrylic 40cm×40cm×40cm chamber as the testing arena for this test. Three 7.5×5 cm (diameter, height) cups made of black aluminum mesh were placed in three corners of the field. Food reinforcers (pea-sized amounts of chocolate-flavored puffed rice) were placed in small weigh boats. The food in two of the weigh boats was covered by the black mesh cups so that it was not accessible to the animal; in the third cup (the “target” cup), the food was accessible and could be consumed. A cotton-tipped laboratory swab, attached to the corner-facing portion of each cup, was extended vertically 5cm from the surface of the cups. Odorants (McCormick flavor extract, USA) were prepared fresh on test day at a 1∶100 dilution. Immediately before each trial, swabs were dipped in the odorant solution to ensure strong olfactory stimuli for the animal. The coffee odor was always associated with the target food cup.

On day one, food was removed from the animals’ home cage. On day two (acclimation day) the food-deprived animals were placed in the test arena for 20 minutes with no food cups present. At the end of that day’s light cycle, animals were introduced to the novel reinforcer in their home cage (10 pea-sized amounts of chocolate-flavored puffed rice). On day three (test day) animals underwent four trials in the field with the three food cups present. On the first trial, the reinforcer was available in a double-portion (two pea-sized amounts) to the animal in the cup marked by the coffee odor. This was to ensure that the food-deprived animal learned to associate the odor with the accessibility of food. The trial persisted until the animal retrieved and consumed the food from the cup. Once the animal completed the trial, it remained in the field for an additional 20 seconds and then returned to its home cage for a six minute inter-trial interval (ITI). The location of the food cups were rearranged on trials 2–4 but the baited “target” cup remained consistently marked by the coffee odor. Both the corner location of the coffee odor and its position relative to the other odors were changed each trial.

On each trial the time (latency) to retrieve and consume the food and number of errors, were live-recorded by the experimenter. An error was defined as any time that an animal made contact with an incorrect cup or attempted to poke inaccessible food with its nose. An error was also recorded when an animal sampled the “target” food but did not consume it. Errors served as the dependent analysis to circumvent the complication of differences in the speed of locomotion of each animal.

#### Marble Burying

This is a test which measures stereotyped, repetitive behavior in mice. Clean home cages were filled with 4 cm of cage bedding. Twenty black glass marbles were placed in five columns of four rows at evenly spaced intervals. The animal was placed in the clear plastic cage with the evenly spaced marbles. Animals were allowed 30 minutes in the cage and the number of marbles buried was recorded. We took several measurements of the percentage of the marble that was buried in the cage. The measurements of 50, 75, 100, and completely buried correspond to the approximate percent of the marble that was buried. The measurement 100% refers to a marble that was buried but some of it can be seen by the experimenter. The measurement completely buried refers to marbles that could not been seen by the experimenter when recorded. We used several measurements to determine which one might be the most sensitive.

#### Fear Conditioning

Utilizing a two-day protocol, animals were placed in an operant-chamber housed within an isolation cubicle for the first day of testing (Coulbourn Instruments, Allentown, PA, USA) which prevented external light or sound from entering the operant-chamber. The operant-chamber was illuminated by an interior light providing constant luminescence (2 lux) and contained a shock-grid floor and a speaker. On day one, animals were placed in the chamber and two minutes of baseline activity was recorded. Then a conditioning stimulus (CS) (85dB white-noise tone) was presented for 20 seconds. At the end of the CS, a two second unconditioned stimulus (US) (.75 milliamp mild foot shock) was presented. There were two CS-US pairings per animals the first day.

To assess this form of amygdala-dependent learning, we used a novel context minus foot shock on day two. In the novel context, a clear acrylic square was placed over the shock grid (proving a novel tactile context) and 1 ml of pure vanilla extract (Adam’s Extracts, USA) was placed in the chamber, beneath the floor (novel olfactory context). On day two, we first recorded the animal’s freezing in the new context for 3 minutes. We then presented the CS for 3 minutes. Throughout all testing the freezing behavior was recorded by the software but an experimenter was present to confirm that the CS was administered to the subject.

### Statistics

The behavioral data with a single measurement were analyzed using an independent samples t-test. The behavioral data with repeated measures were analyzed using a two-way ANOVA with experimental group as the independent factor and the trials or block number as the repeated factor. All data were analyzed using SPSS 20.0 (IBM, USA). Values are shown as mean ± S.E.M. for each group.

## Results

### Aniracetam does not alter activity levels or anxiety-like performance in the open field test

**Figure 1:**
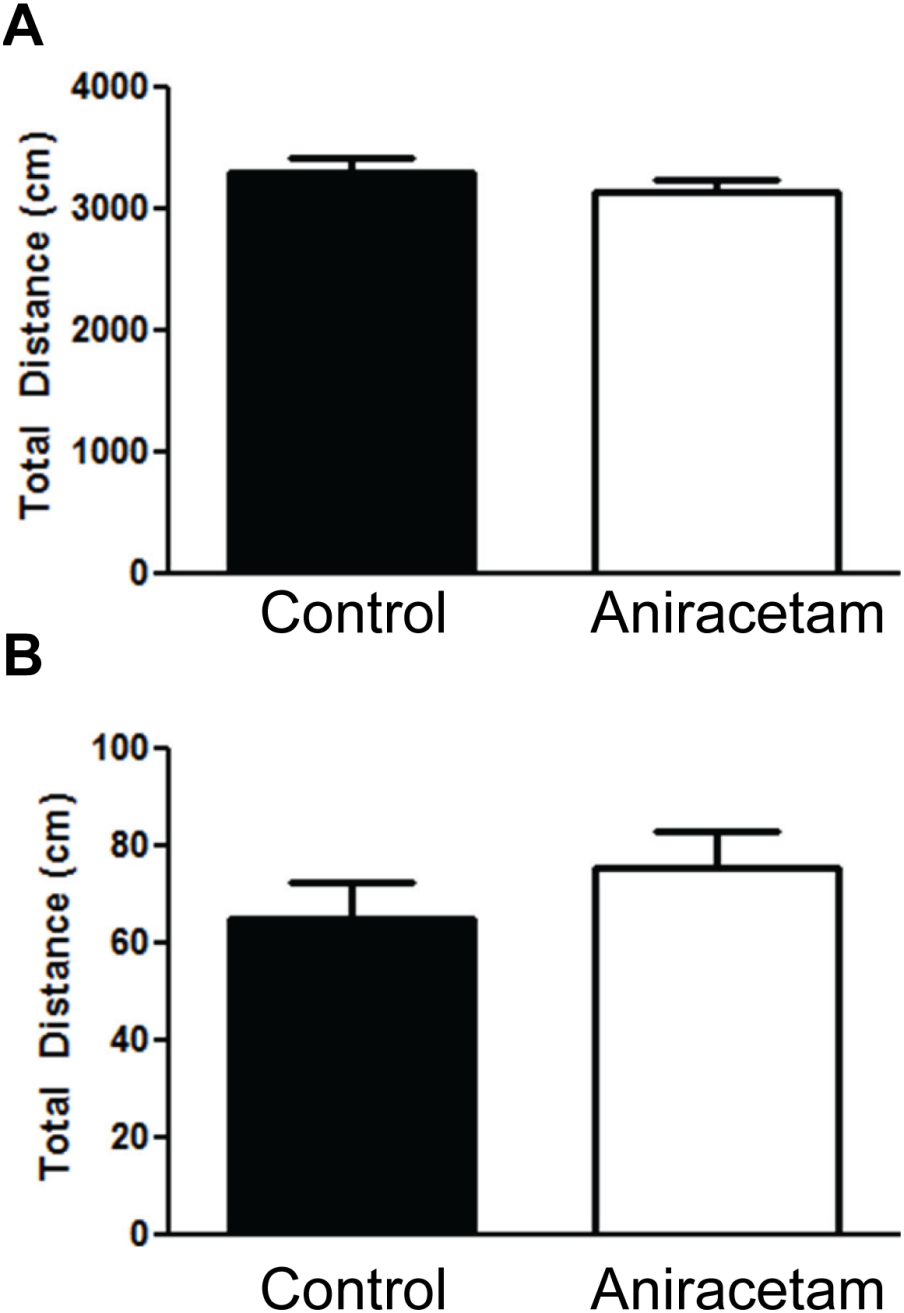
Aniracetam treatment did not alter locomotion in the open field. (A) Control mice and mice given aniracetam did not show a difference in total distance moved in an open field test. (B) Control mice and mice given aniracetam did not show a difference in total distance moved in the center of the open field test. Graphs show the mean (± SEM). n = 15 per group.

There were no differences in locomotion or anxiety between the control group and the aniracetam-treated group in the open field test. An independent t-test found no difference between the groups in total distance moved in the 10 minute trial t(1,28) = 1.05, p = 0.3 (Figure 1A). An independent t-test found no difference between the group in total distance moved in the center of the open field test t(1,28) = 0.99, p = 0.33 (Figure 1B). There was no difference in number of fecal boli produced in the open field test with 1.73±0.43 (mean ± standard error of the mean) for the control group and 1.8±0.34 for the experimental group.

### Aniracetam does not alter performance in the elevated plus maze test

**Figure 2:**
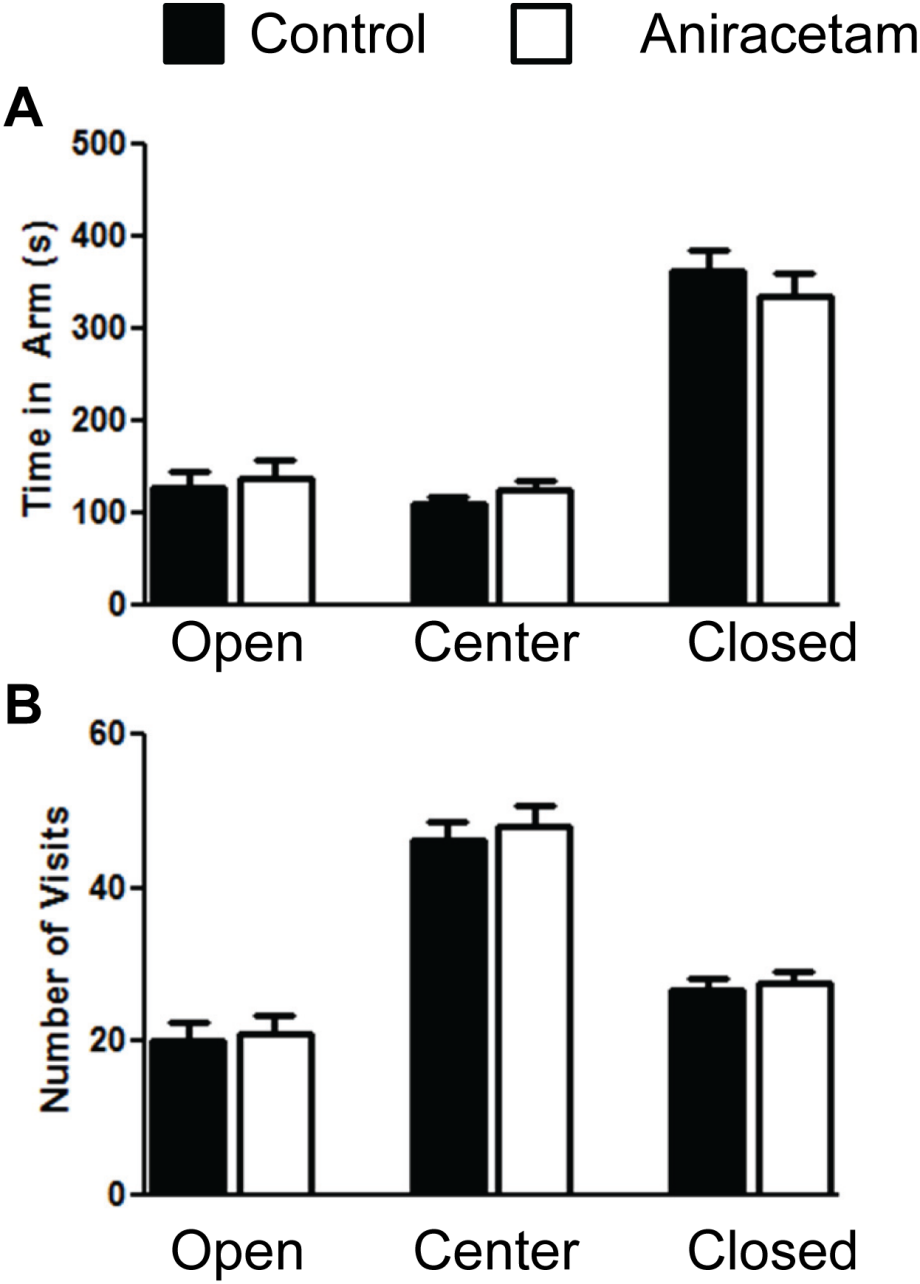
Aniracetam treatment did not alter performance in the elevated plus maze test. (A) Control mice and mice given aniracetam did not show a difference in time in the open, center, or closed arms in the 10 minute trial in the elevated-plus maze test. (B) Control mice and mice given aniracetam did not show a difference in number of visits in the open arm, center arm, or closed arm in the 10 minute trial in the elevated-plus maze test. Graphs show the mean (± SEM). n = 15 per group.

We then examined whether Aniracetam treatment altered performance in the elevated plus maze test. It has previously been reported that aniracetam treatment alters anxiety in three different models of anxiety. [12]. The models include social interaction test, elevated-plus maze test, and conditioned fear stress tests. We found no difference in time or in the number of visits in the open arms, center, or closed arms. An increase in time in the open arms would have suggested a decrease in anxiety. An independent measures t-test found no difference in time in the open arm t(1,28) = 0.44, p = 0.66; center arm t(1,28) = 1.23, p = 0.23; or closed arms t(1,28) = 0.86, p = 0.40 (Figure 2A). We found similar results in the frequency in each arm with no difference in time in the open arm t(1,28) = 0.22, p = 0.83; center arm t(1,28) = 0.44, p = 0.66; or closed arms t(1,28) = 0.43, p = 0.67 (Figure 2B). We separately analyzed the distance moved in the elevated plus maze test and found no difference in total distance moved t(1,28) = 1.06, p = 0.30. One interesting finding is that there was a difference in the number of fecal boli produced in the ten minute plus maze t(1,28) = 2.08, p<0.05. The number of fecal boli produced was 1.28±0.48 (mean ± standard error of the mean) for the control group and 0.73±0.31 for the experimental group. However, we did not find this difference in the open field test.

### Aniracetam does not alter motor learning

**Figure 3:**
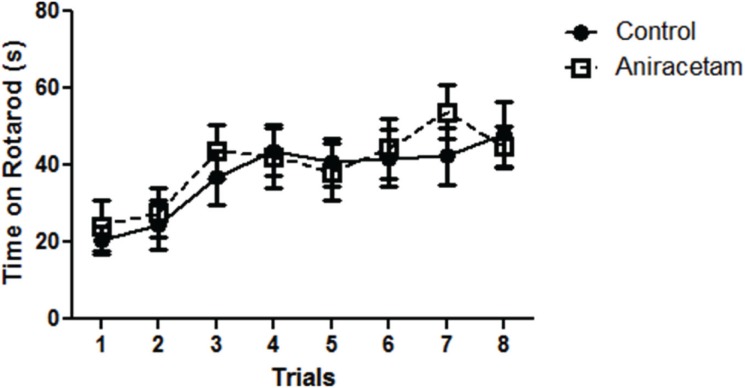
Aniracetam treatment did not alter motor learning in the accelerating rotarod test. (A) Control mice and mice given aniracetam did not show a difference in their ability to remain on an accelerating rotarod across 8 trials. Graphs show the mean (± SEM). n = 15 per group.

We used an accelerating rotarod test to examine differences in motor learning after aniracetam treatment and found no difference in motor learning between the groups over the 8 trials (Figure 3). We used a two-way ANOVA to examine differences between the two groups over the 8 trials and did not find a difference between the groups F(1, 28) = 0.10, p = 0.75. The within-subjects analysis did not reveal a group×trial interaction F(7,196) = 0.69, p = 0.68. However, there was a main effect of trial F(1,7) = 9.69, p<0.001. Therefore, both groups did demonstrate improvement in their ability to stay on the accelerating rotarod across the eight trials, but there was no difference between the two groups over the trials.

### Aniracetam does not alter spatial learning and memory

**Figure 4:**
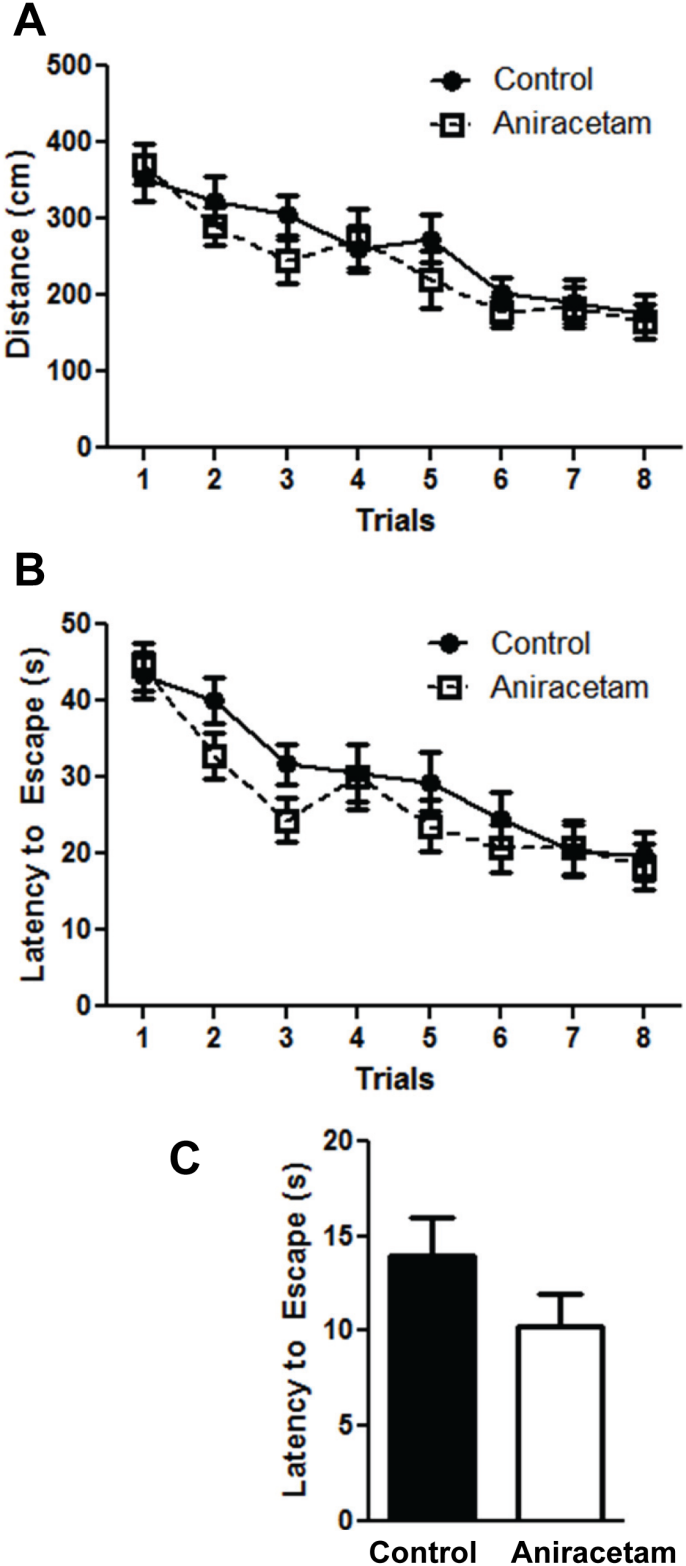
Aniracetam treatment did not alter spatial navigation in the Morris water maze test. (A) Control mice and mice given aniracetam did not show a difference in their ability to find a hidden platform across the 8 trials. We measured the path length traveled to find the hidden platform. (B) Control mice and mice given aniracetam did not show a difference in their ability to find a hidden platform across the 8 trials. We measured the latency to find the hidden platform. (C) Control mice and mice given aniracetam did not show a difference in their ability to find a visible platform. Graphs show the mean (± SEM). n = 15 per group.

The Morris Water Maze is a classic test used to measure spatial navigation. We used the Morris water maze test to evaluate the possible cognitive enhancing effects of aniracetam on hippocampus-dependent learning and memory. We have previously published results using this protocol to investigate learning and memory changes in mice [16]. We did not observe any change in learning between the control and drug treatment across the learning trials. There was no difference in distance traveled between the control and drug treated group F(1,28) = 0.89, p = 0.35 (Figure 4A). There was no interaction between group and trial F(7,196) = 0.59, p = 0.76. However, both groups showed improvement in their ability to find the hidden platform across the eight trials F(7,196) = 2.64, p<0.001. We found similar results when we examined the latency to find the hidden platform across the 8 blocks. There was no difference between the groups in their latency to find the hidden platform F(1,28) = 1.21, p = 0.28 (Figure 4B). There was no interaction between group and trial F(7,196) = 0.74, p = 0.63. However, both groups showed improvement in their ability to find the hidden platform across the eight blocks F(7,196) = 3.93, p<0.001. The results from the distance to find the hidden platform and latency to find hidden platform suggest that aniracetam treatment does not improve spatial navigation in mice. We examined whether there was a difference in the ability to find a visible platform. There were no differences between the group in terms of distance to reach the visible platform t(1,28) = 1.0, p = 0.32; or in the latency to find the visible platform t(1,28) = 1.45, p = 0.15 (Figure 4C). One caveat to our testing protocol is that we did not include a probe test after the last block of testing. We did not include the probe test in order to complete all testing within the 3 hour therapeutic window of aniracetam treatment. The probe trial involves a trial where the hidden platform is removed and the subject is allowed to explore the empty maze for 1 minute [18]. The time the animal spends in each quadrant and the number of visits per quadrant is measured. If the animal remembers the location of the hidden platform it should spend more time and visit the quadrant that previously held the hidden platform. This is an important measure of spatial memory and increased performance in this test would have implied that even though aniracetam did not improve learning it did result in enhanced spatial memory.

### Aniracetam does not alter repetitive behavior

**Figure 5:**
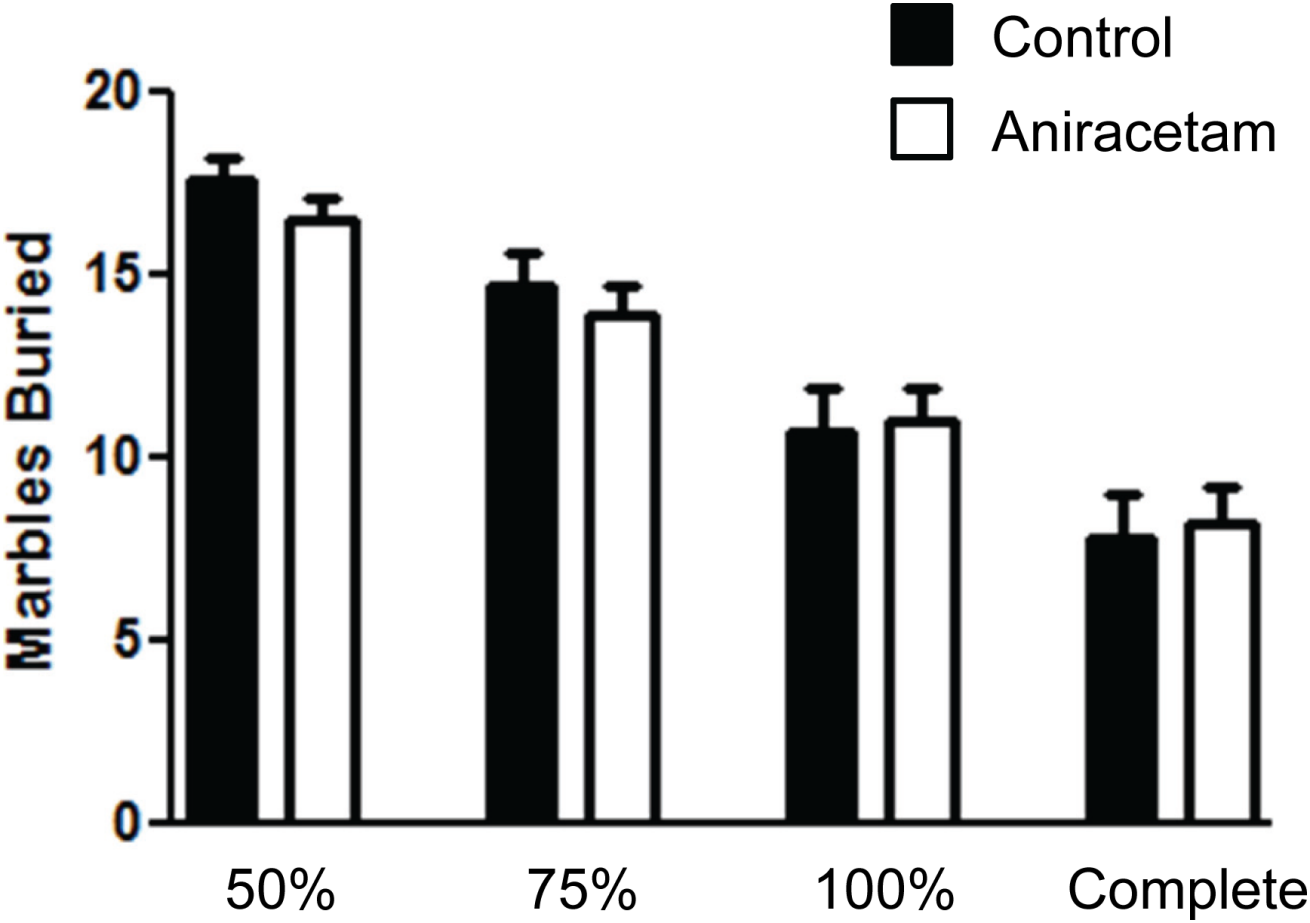
Aniracetam treatment did not alter repetitive behaviors in the marble burying test. (A) Control mice and mice given aniracetam did not show a difference in their ability to bury marbles in a 30 minute test trial. Graphs show the mean (± SEM). n = 15 per group.

We used the marble burying test to determine whether aniracetam altered repetitive behaviors. We did not observe any statistical differences between the groups across the different measures of marbles buried (Figure 5). There was no difference in the animal’s performance in burying the marble when measured at: 50% t(1,28) = 1.2, p = 0.24; 75% t(1,28) = 0.57, p = 0.57; 100% t(1,28) = 0.17, p = 0.86; or at the level of completely burying them so that they were not visible t(1,28) = 0.25, p = 0.80. It does not appear that treatment with aniracetam altered repetitive behavior in mice.

### Aniracetam does not alter fear conditioning

**Figure 6:**
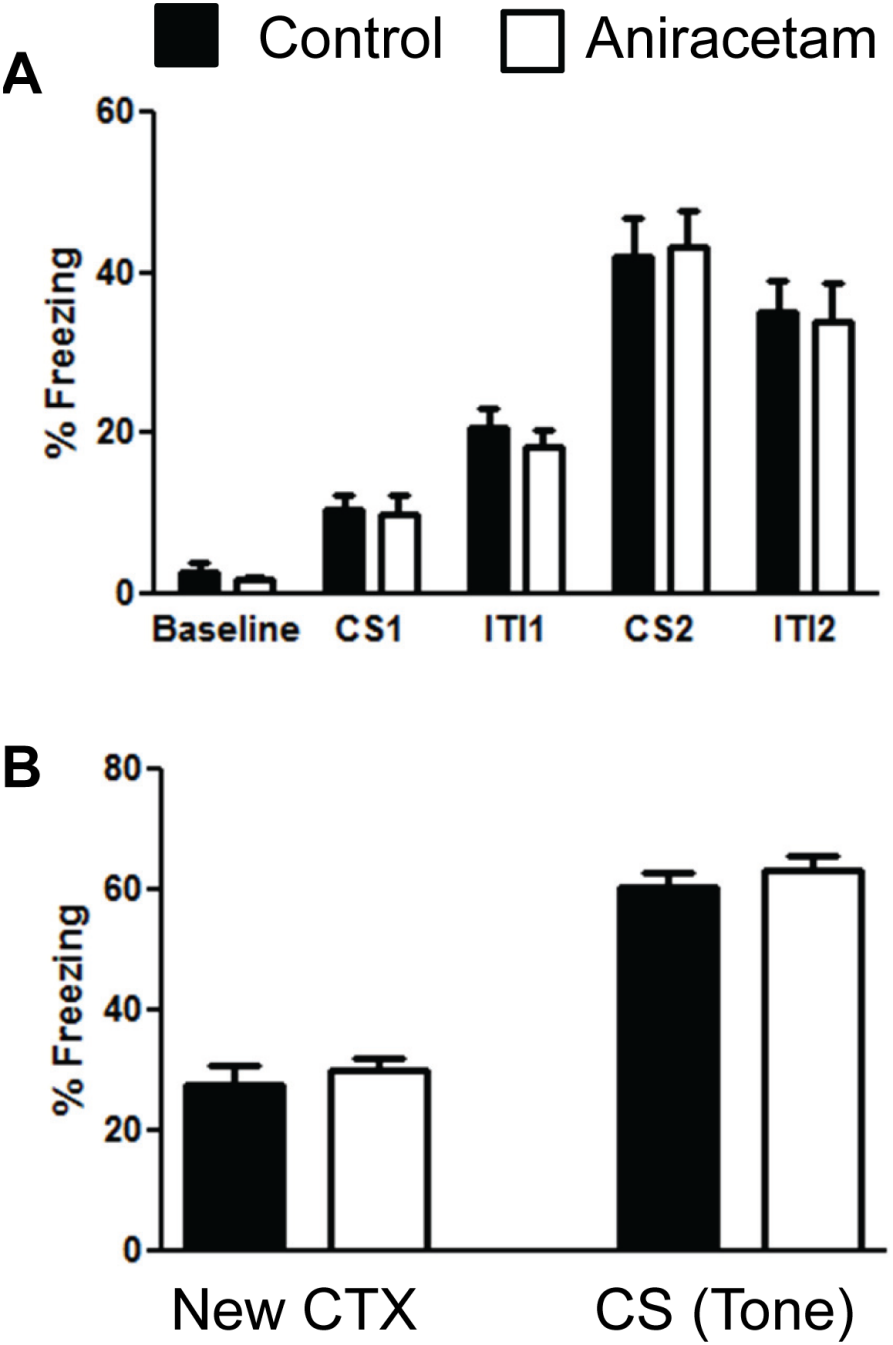
Aniracetam treatment did not alter fear conditioning. (A) Control mice and mice given aniracetam did not show a difference in their ability to learn to associate a novel tone with an aversive stimulus (mild shock). (B) We then examined their ability to remember this association 24 hours later. There was no difference between the groups in terms of freezing in a novel context and there was no difference in freezing to the conditioned stimulus when it was presented over the 3 minute trial. Graphs show the mean (± SEM). n = 15 per group.

The main objective of this study was to determine the possible cognitive enhancing effects of aniracetam. The rotarod test examined motor learning and the Morris Water Maze examined spatial navigation. We used fear learning to determine associative conditioning changes after aniracetam treatment. In this test, the animal was presented with a tone which was then paired with an aversive stimulus (shock). Within two presentations the mice will form an association between the tone and the shock. Therefore, when presented with the conditioned stimulus (tone) the mice should show an increase in freezing in anticipation of the shock. We did not observe any difference in the ability of the mice to learn to associate the tone with the shock on day one of conditioning (Figure 6A). There was no main effect of group across the different conditioning trials F(1,28) = 0.08, p = 0.77. There was no interaction between group over the different testing parameters F(4,112) = 0.11, p = .98. There was a main effect of learning over the trials F(28, 112) = 2.14, p<0.01. Therefore, both groups did show increased freezing throughout the first day of conditioning. We then examined their freezing levels when presented in a novel context for three minutes and found no difference in freezing t(1,28) = 0.64, p = 0.52 (Figure 6B left graph). When we presented the conditioned stimulus in the new context both groups showed an increase in freezing to the tone. However, there was no difference between the groups in the amount of freezing when presented with the tone t(1,28) = 0.86, p = 0.39 (Figure 6B right graph). These results demonstrated that treatment with aniracetam did not enhance fear conditioning.

### Aniracetam does not alter odor discrimination learning

**Figure 7:**
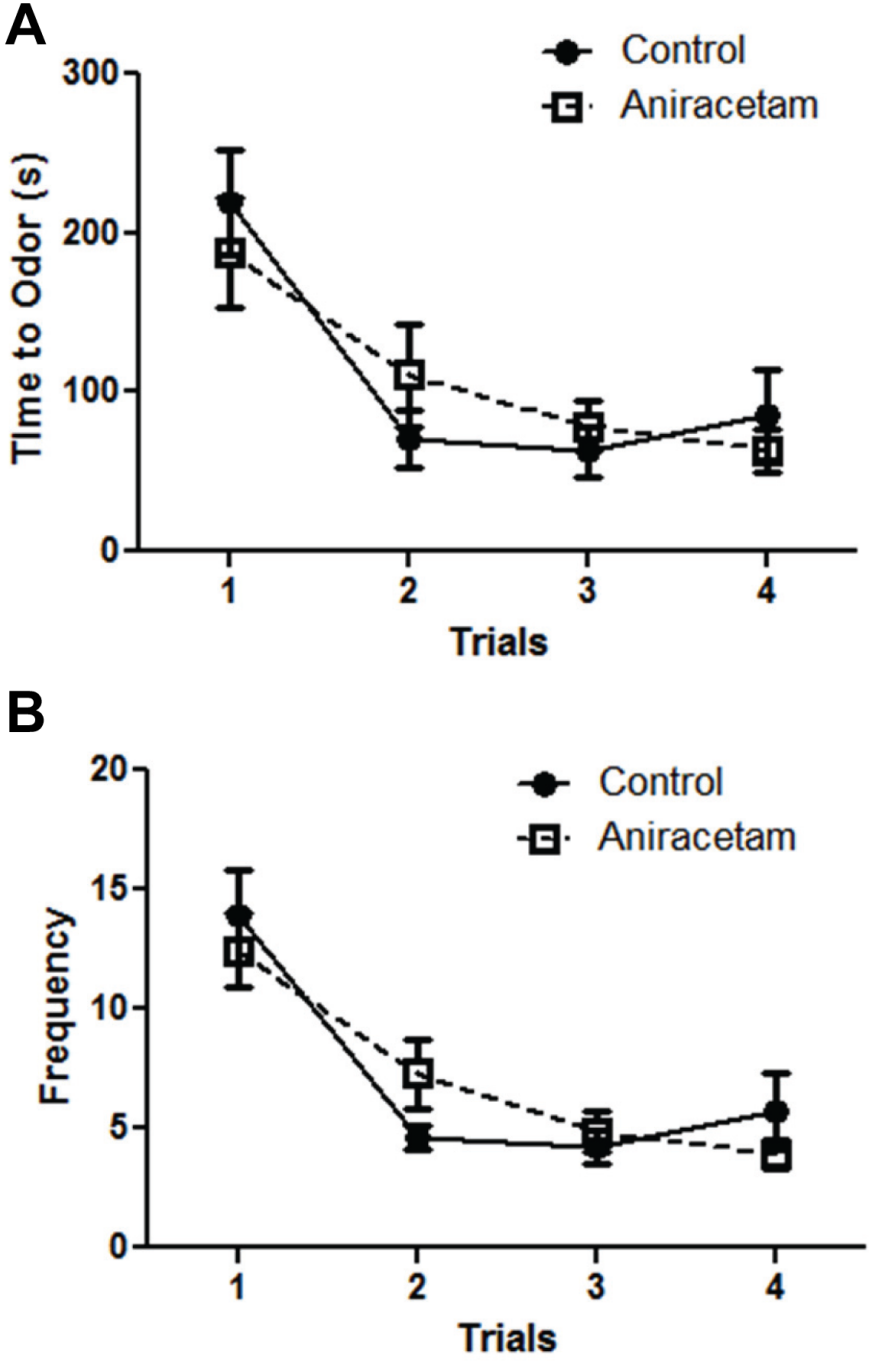
Aniracetam treatment did not alter learning in the odor discrimination test. (A) We used odor discrimination to probe. The dependent measures were the time to target odor and the frequency of errors (errors were defined as interacting with the wrong cup and encountering but not eating from the target cup. There was no significant difference between the learning curves of the control and drug groups (**A** and **B**).

We did not find any differences between the groups in odor discrimination learning (Figure 7A). We used a two-way ANOVA and found no main effect of group F(1,27) = 0.001, p = 0.98 for the effect over time. There was a main effect of trial F(3,81) = 14.12, p<0.001. There was no group×trial effect F(3,81) = 0.98, p = 0.41. Similar results were found when investigating frequency of errors over time (Figure 7B). We used a two-way ANOVA and found no main effect of group F(1,27) = 0.001, p = 0.98 for the effect over time. There was a main effect of trial F(3,81) = 21.55, p<0.001. There was no group×trial interaction F(3,81) = 1.4, p = 0.25.

## Discussion

We demonstrate that aniracetam does not enhance cognitive performance in healthy subjects across a variety of behavioral tasks that measure spatial learning, fear learning, and motor learning. In addition, aniracetam does not influence performance in the elevated plus maze, locomotion, or repetitive behaviors in C57BL/6J mice. We included these additional tests to determine if aniracetam results in other behavioral changes that could influence learning and memory in mice. Our studies clearly demonstrate that repeated doses of 50 mg/kg of aniracetam presented orally does not produce changes in learning and memory, performance in tests that measure anxiety, locomotion, or repetitive behavior.

Our initial hypothesis was that aniracetam treatment would result in enhanced learning and memory based on previous studies. Aniracetam positively impacts the pharmacological profile associated with learning and memory by elevating hippocampal acetylcholine, serotonin, glutamate, and dopamine levels [12], [19], [20]. In addition, aniracetam significantly facilitates long-term potentiation (LTP) formation in the hippocampus [21], reverses memory loss [22], reduces anxiety [12], and reverses ethanol-induced brain damage [23], [24]. An important caveat for some of the above behavioral studies is that they were performed in rodent models of disease or using experimentally induced learning deficits and did not explicitly address impacts in healthy subjects. For instance, one study found that a 50 mg/kg oral dose of aniracetam treatment reverses learning and memory deficits in rats that were previously injected with scopolamine then tested in a passive avoidance test [22]. This study did not examine whether aniracetam without scopolamine treatment enhances learning and memory. In another study, rats were exposed to ethanol during prenatal development [24]. During the early postnatal period the rats were given 50 mg/kg (intubated by gavage) treatment over 10 days. The aniracetam treatment reversed learning and memory deficits in an active avoidance task, but did not improve cognitive performance in control rats. Even though these studies did not explicitly examine whether aniracetam has cognitive enhancing properties, the lack of enhanced performance in control subjects does provide evidence that aniracetam does not improve learning in healthy subjects. Our investigation, to our knowledge, was the first to comprehensively study the effects of aniracetam across a variety of behaviors in healthy subjects.

There are several possible reasons why we did not observe alterations in behavior. One reason may be due to the time frame used in our study. In a previous study, aniracetam was infused into the hippocampus. They found that intrahippocampal infusion of 2.0 to 4.0 mM of aniracetam enhances basal synaptic transmission in the dentate gyrus but the effect is only present for 30 minutes [21]. The investigators found improvement in rodent learning in the Y-maze but their studies are also performed within this narrow window of treatment. The investigators reported that the effects of aniracetam on LTP in the hippocampus recovered to control level within 1 hour. It may be that aniracetam only has a 30 minute therapeutic window for improving learning and memory. In addition, the investigators found that aniracetam did not increase the ceiling for LTP induction. They found that rats with aniracetam treatment reached the maximal LTP induction more quickly. A future study could use passive avoidance to investigate the impact of aniracetam on one-trial learning. It may be that the effect of aniracetam is only observed at the early stages of learning and may have its strongest impact during this phase of learning. However, aniracetam treatment did not appear to improve fear conditioning on the initial learning day. There was not an increase in freezing in the mice given aniracetam on the second trial of fear conditioning (Figure 6A). Even though this is not a sensitive test of one-trial learning an increase in freezing in the aniracetam group on the conditioning day would have suggested improved acute learning.

Another study, which examined the pharmacokinetics of aniracetam in rats, found that oral administration (50 mg/kg) of aniracetam attains peak levels within 30 min post-dosing [25]. In our study, we began testing within 1 hr after the dose was administered to the animals. The C_max_ after oral administration (50 and 100 mg/kg) occurs at 20 min after dosing using HPLC. The plasma concentration from oral administration (50 mg/kg) was approximately 1 µg/ml, which quickly decreased. However, the metabolites of aniracetam remain much higher in the plasma concentration for many hours. There was some inconsistency with a previous study which administered 50 mg/kg of [^14^C] Aniracetam orally to rats and found that the half-life was 2.1 h in males and 1.7 h in females [26]. Despite the inconsistent results the 50 mg/kg dose has been previously examined and does appear to be present in the plasma quickly after administration. Future studies could administer aniracetam and investigate learning and memory changes in tests such as novel object recognition and fear conditioning within 30 minutes of administration.

Another reason why we did not observe alterations in behavior with aniracetam treatment may be the dosage used in our study. In a previous study, the investigators presented the animals with 30 mg/kg and 100 mg/kg of oral administration of aniracetam [19]. They found a significant increase in dopamine, serotonin, and their metabolites in the prefrontal cortex, dorsal hippocampus, and basolateral amygdala. In most regions the elevation lasted more than 180 minutes. One caveat of the study is that the elevation is seen in rats that are considered an animal model of multiple infarction. Since this is a disease model, it is not clear whether such changes occur in healthy rats. However, several studies have used a 50 mg/kg dose and found that this dose reverses learning and memory deficits [22], [24] and oral 50 mg/kg dose of aniracetam reverses impaired AMPA receptor mediated transmission in the hippocampus of rats prenatally exposed to ethanol [23], [27]. It is possible that a higher dose is necessary to result in improvement in learning and memory in normal healthy mice. However, we wanted to use a dose that has repeatedly been demonstrated to be effective in some experimental models. One study found that oral administration of 25 mg/kg and 100 mg/kg of aniracetam did not restore object recognition in 16- to 18- month old rats while 50 mg/kg did restore object recognition [7]. The effect of oral administration of 50 mg/kg lasted for four hours in the aged rats. The authors suggested that an inverse U dose-effect relationship occurs in aniracetam for object recognition since the lower and higher doses were not effective. Based upon these studies we determined that oral administration of 50 mg/kg would be the dose most likely to improve learning and memory. Future studies could examine the influence of a 100 mg/kg dose in mice and determine the influence of this dose on learning and other behavioral features.

The evidence is strong that aniracetam is effective for enhancing cognitive performance in impaired (i.e. brain damaged) subjects but appears to be ineffective in healthy subjects. However, it is difficult to examine a single mechanism that could underlie the positive effects of aniracetam. Aniracetam has been shown to decrease membrane fluidity in the hippocampus and frontal cortex of aging mice [28]. The authors of this paper believed that such action could reverse some of the effects associated with Alzheimer’s disease. In a separate paper, investigators administered scopolamine to induce a learning deficit [7]. They found that aniracetam given to aging rats results in a restored ability for object recognition. They hypothesized that the restoration may be due to improvement of the cholinergic system. Despite the lack of consensus on the mechanism of action for aniracetam, it does seem effective in improving learning and memory after the animals have had experimentally induced damage to the brain. However, it does not appear that aniracetam alters behavior when presented at 50 mg/kg to normal healthy mice.

## Conclusion

Even though the results we presented here are negative results we do believe that they are useful for other investigators. We took the approach of administering aniracetam orally to mimic the most common method that humans would use and used a dose that has been frequently used. In addition, we examined a number of aspects of cognitive behavior and measured other behaviors that may be altered with anireactam treatment. Our behavioral measures did not provide any evidence that aniracetam is an effective cognitive enhancer. If other investigators are interested in the cognitive enhancing properties of aniracetam then they may use our results as a starting point and make alterations to the experimental design for future studies.

## Supporting Information

Checklist S1 ARRIVE checklist.
(DOC)
